# The mitigatory capabilities of exercise on breast cancer chemotherapy-induced cardiotoxicity

**DOI:** 10.3389/fcell.2025.1681702

**Published:** 2025-11-07

**Authors:** Chen-yue Qian, Juan Fan, Jing-yi Guo, Na Li, Xiang-qi Liu, Xiu Liu, Xiang-yuan Zeng, Cui-feng Huang, Cong Li, Hua-dong Liu, Jing-jin Liu

**Affiliations:** 1 Department of Intensive Care Unit, Southern University of Science and Technology Yantian Hospital, Shenzhen, Guangdong, China; 2 The Second Clinical Medical College, Jinan University, Shenzhen, Guangdong, China; 3 Department of Geriatrics, Shenzhen People’s Hospital (The Second Clinical Medical College, Jinan University, The First Affiliated Hospital, Southern University of Science and Technology), Shenzhen, Guangdong, China; 4 Department of Radiation Oncology, Shenzhen People’s Hospital, The Second Clinical Medical College, Jinan University, Shenzhen, Guangdong, China; 5 Department of Cardiology, Shenzhen People’s Hospital (The Second Clinical Medical College, Jinan University, The First Affiliated Hospital, Southern University of Science and Technology), Shenzhen, Guangdong, China; 6 Department of Cardiology, Shenzhen Cardiovascular Minimally Invasive Medical Engineering Technology Research and Development Center, Shenzhen People’s Hospital (The Second Clinical Medical College, Jinan University, The First Affiliated Hospital, Southern University of Science and Technology), Shenzhen, China; 7 Shenzhen Key Laboratory of Stem Cell Research and Clinical Transformation, Shenzhen People’s Hospital (The Second Clinical Medical College, Jinan University, The First Affiliated Hospital, Southern University of Science and Technology), Shenzhen, Guangdong, China

**Keywords:** breast cancer, chemotherapy, cardiotoxicity, exercise, cardiac rehabilitation

## Abstract

Chemotherapy drugs have significantly decreased breast cancer morbidity and mortality, but they have been associated with increased risk for adverse cardiovascular side effects, such as cardiotoxicity. These drugs generally fall under three broad categories: cell cycle inhibitors (ex. Anthracyclines, taxanes), human epidermal growth factor 2 (HER2) blockers (ex. Trastuzumab, pertuzumab), as well as other medications, such as the alkylating agent cyclophosphamide. This review analyzes the occurrence of specific cardiotoxic manifestations linked to increased heart failure risk, such as chest discomfort, edema, and dyspnea, as well as mechanisms of action, such as anthracycline inducing the generation of reactive oxygen species, for the aforementioned three drug categories. In particular, attention is given to anthracyclines and HER2 blockers, as they are two of the most commonly prescribed medications. On the other hand, the cardiotoxic effects of these medications have been found to be able to be mitigated by various exercise regimens, such as aerobic exercises, resistance training, and high intensity interval training. This review also examines the effectiveness of different regimens on alleviating post-chemotherapy cardiotoxicity in breast cancer patients, as well as the specific mechanisms involved, such as aerobic exercise being able to downregulate the expression of doxorubicin-induced pro-inflammatory factors (ex. Interleukin-8, cyclooxegenase-2, etc.). Moreover, the review points out the relative lack of cardiac rehabilitation programs specifically addressing the post-chemotherapeutic cardiotoxicity risks of breast cancer patients. Therefore, customized exercise regimens, accounting for breast cancer patient-specific medical profiles, should be developed to counteract against the adverse cardiovascular effects of chemotherapy.

## Introduction

Breast cancer is one of the most widespread cancers worldwide, and a leading cause of death among women (Momenimovahed and Salehiniya, 2019). At least four distinct clinically significant molecular subtypes of breast cancer are thought to exist since the seminal work of Perou, Sørlie, and associates at the start of this millennium: basal-like, HER2-enriched, luminal A, and luminal B (Perou et al., 2000; Sørlie et al., 2001). Consequently, to treat the disease, customized multimodal strategies, involving surgery, radiation, as well as chemo-, targeted, and endocrine therapies, are essential (Gillespie et al., 2011). Such strategies are all influenced by breast cancer stage, grade, and molecular subtype, and selecting more effective treatment options, particularly in the form of focused therapies, have been linked to higher survival rates (Omland et al., 2022; Curigliano et al., 2016). With respect to chemotherapy, they fall into three broader categories: cell division inhibitors (anthracyclines (Monsuez et al., 2010; Narezkina and Nasim, 2019), taxanes, etc.), human epidermal growth factor 2 (HER2) blockers (ex. Trastuzumab, pertuzumab, etc.) (Eaton and Timm, 2023), as well as other agents, such as the alkylating agent cyclophosphamide and anti-metabolite fluorouracil (5-FU) (Valiyaveettil et al., 2023; Krop et al.; Moslehi, 2016). Their development has increased overall long-term breast cancer survival rates, but they are also associated with cardiotoxicity, which is considered to be a significant cause of breast cancer patient mortality (Agha et al., 2022). However, the precise association between chemotherapy and cardiotoxicity, as well as their associated pathological processes, have not been precisely defined. More specifically, chemotherapy-related cardiotoxicity (CRCT) not only entails the direct effects of chemotherapy on the entire cardiovascular system, but also indirect ones, stemming from changes in thrombogenic states or hemodynamic flow (Albini et al., 2010). Ultimately, CRCT, by interfering with cancer treatments, could result in congestive heart failure (HF) during or post-chemotherapy, thereby lowering patient survival rates and quality of life (Oikawa et al., 2023; Piepoli et al., 2022). Indeed, according to the 2022 cardio-oncology recommendations from the European Society of Cardiology (ESC), cardiotoxicity entails cardiac dysfunction, myocarditis, vascular toxicity, arterial hypertension, and arrhythmia. In particular, cardiac dysfunction has been found to account for 48% of cardiotoxicity occurrences in cancer patients, and can be divided into two types: asymptomatic, which is identified by measuring myocardial global longitudinal strain, pathological cardiac biomarkers, and left ventricular ejection fraction (LVEF), as well as symptomatic, which is characterized by ankle edema, dyspnea, and exhaustion; furthermore, symptomatic cardiac dysfunction is considered a sign of HF (Lyon et al., 2022). The ESC recommendations highlight echocardiography’s fundamental role in baseline evaluation and ongoing surveillance. Its primary measurements are global longitudinal myocardial strain (GLS) and left ventricular ejection fraction (LVEF). A substantial drop in LVEF is typically preceded by a relative fall in GLS, which is thought to be an early sensitive indicator of subclinical ventricular dysfunction. The guidelines recommend the measurement of B-type natriuretic peptide (BNP) or N-terminal pro-BNP (NT-proBNP) for the assessment of hemodynamic stress, as well as the continuous measurement of high-sensitivity cardiac troponin (hs-cTn) for the detection of persistent myocardial injury. The rise in these biomarkers, particularly troponin, can help determine which individuals are more likely to experience a subsequent decrease in LVEF and direct the start of cardioprotective therapy (Lyon et al., 2022).

Common CRCT-linked symptoms, observed among breast cancer patients, include tiredness, palpitations, peripheral edema, chest discomfort, and dyspnea. In particular, chest discomfort, edema, and dyspnea are common among patients on HER2 blockers and/or anthracycline-based medications, though it is worth noting that the development of those HF-related symptoms manifested later than patients who were on capecitabine (Kim et al., 2024). This may be due to HER2 blocker- or anthracycline-based regimens being associated with more gradual cardiomyocyte and other myocardial cell damage (Anjos et al., 2021). These symptoms may be caused by deterioration in ventricular filling or ejection processes (Shams e t al., 2024). This observation is also supported by Salyer et al. (2019), who examined the clustering of common HF symptoms, which generally fell into three groups: gastrointestinal disruption, illness-related discomfort, and sickness behavior. Pain, edema, and dyspnea was found to be part of the illness-related discomfort cluster, which was consistent with the symptoms previously observed among patients receiving HER2 blockers and/or anthracyclines (Salyer et al., 2019). Therefore, patients receiving such chemotherapeutic treatments should be monitored for HF-linked signs and symptoms, such as chest discomfort, edema, or dyspnea. Aside from HER2 blockers and/or anthracyclines, CRCT could also be found among patients taking antimetabolite regimens, such as oral capecitabine, in which a predominant symptom is vasospasm-caused chest pain, plus vasospasm-related arrhythmia, myocardial disease, and ischemia (Padegimas and Carver, 2020). In fact, previous studies have reported that patients taking the antimetabolite drugs 5-fluorouracil, or capecitabine, demonstrated chest pain at up to 72 h after the first administration of these drugs (Padegimas and Carver, 2020; Kanduri et al., 2019; Garbis et al., 2023; Dyhl-Polk et al., 2020). It is worth noting, though, that aside from different chemotherapy regimens, vasospasm could also be caused by reactive oxidative stresses, endothelial dysfunction, and hypersensitive vascular smooth muscle (Sheth et al., 2021; Hokimoto et al., 2023). Overall, numerous breast cancer chemotherapeutic drugs, such as anthracyclines and taxanes, as well as molecular-targeting drugs, like trastuzumab and pertuzumab, could potentially cause cardiotoxicity. The cardiotoxic effects of each group of drugs are described below.

### Breast cancer treatment agents associated with cardiotoxicity

Chemotherapy for breast cancer is primarily classified as adjuvant/neoadjuvant chemotherapy and metastatic treatment, depending on the disease stage and target. Adjuvant chemotherapy for early-stage breast cancer seeks to eradicate any remaining micrometastases following surgery, whereas neoadjuvant chemotherapy is used to reduce the tumor size in preparation for surgery or breast preservation. Chemotherapy is mostly palliative for metastatic breast cancer, with the goals of controlling the illness, reducing symptoms, and extending survival (Guidelines Presidium Lecture Tour, 2022). Cancer molecular typing is used to choose chemotherapy medications. The mainstays of treatment for HER2-negative breast cancer are taxanes (paclitaxel and docetaxel) and anthracyclines (doxorubicin and epirubicin), which are frequently administered one after the other or in combination (Group et al., 2012). Chemotherapy and HER2 blockers (such trastuzumab and pertuzumab) are the usual treatment for HER2-positive breast cancer since they greatly enhance prognosis (von Minckwitz et al., 2017). Platinum-based medications (such carboplatin) have shown exceptional success for triple-negative breast cancer or certain situations (Tutt et al., 2021). Furthermore, the therapeutic landscape has been transformed by antibody-drug conjugates (such T-DM1 and DS-8201) can precisely deliver extremely potent cytotoxic medications to cancer cells (Modi et al., 2020). The following will elaborate on various chemotherapy drugs and their effects on cardiotoxicity.

### Anthracyclines

Adjuvant chemotherapy regimens, based on anthracyclines, were first used in the 1960s, and survival rates for these regimens have significantly improved over with past few decades, yielding 20%–30% reductions in mortality likelihood (Group et al., 2012). However, cardiotoxicity is a common side effect for the most widely-used anthracyclines (Abdel-Qadir et al., 2017), occurring within the first year in 98% of instances (Cardinale et al., 2015). Additionally, 4%–36% of individuals, solely taking anthracyclines, may develop chemotherapy-associated heart dysfunction, with 18% having sub-clinical, and 6% clinically overt cardiotoxicity (Srikanthan et al., 2017). Moreover, cardiovascular-associated mortality has been identified as the primary cause of death for breast cancer survivors, who developed HF post-anthracycline treatment (Khouri et al., 2012).

Anthracycline-associated cardiotoxicity was first observed clinically in 1979, among adult cancer patients with congestive HF (CHF), by Von Hoff et al., in which the CHF occurrence exhibited a cumulative dose-dependent association with the anthracycline doxorubicin (DOX), with incidences of 3%, 7%, and 18%, at, respectively, 400, 550, and 700 mg/m^2^ DOX (Hoff et al., 1979). Therefore, CHF likelihood increases with cumulative dosage, despite more recent studies showing that more vigorous dosing regimens (Author AnonymousEarly Breast Cancer Trialists’ CollaborativeGroup EBCTCG, 2023) and larger cumulative doses are more beneficial in treating breast cancer (Dempke et al., 2023). In fact, doses as little as 180 mg/m^2^ caused DOX-induced damage in ¾ of patients, and severity increased with increasing dosages (Friedman et al., 1978). Furthermore,a separate study showed that the suppression of MALAT1 enhanced cell apoptosis and sensitized BC cells to taxanes and adriamycin, bolstering their responsiveness to these drugs (Hussain et al., 2024). Therefore, no safe dosage for administrating DOX is present (Herrmann et al., 2014), though higher-dose anthracyclines (e.g., DOX≥250 mg/m^2^) are considered to be at increased risk for developing cardiac dysfunction (Armenian et al., 2017). Ultimately, taking into account that the most significant risk factor for anthracycline cardiotoxicity is the total cumulative dosage (Manrique et al., 2017), it is strongly advised that total DOX should not be > 550 mg/m^2^.

**FIGURE 1:**
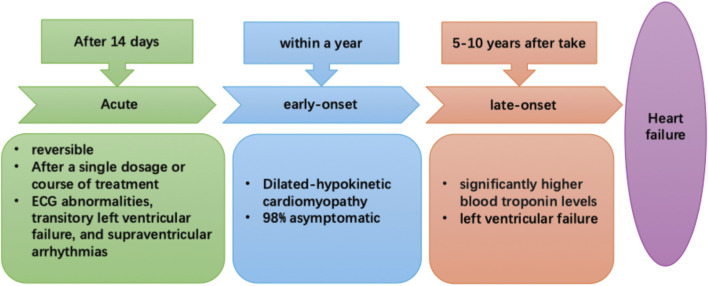
The three types of anthracycline-induced cardiotoxicity: acute (after 14 days), early- (<1-year) and late-onset (5–10 years post-chemotherapy).

Anthracyclines also, by altering DNA structures and blocking their ability to interact with other proteins and enzymes, prevent cell division (Broder et al., 2008). Furthermore, they have been linked to the generation of reactive oxygen species (ROS) and inflammatory mediators; ROS, due to them inhibiting cardiomyocyte functions, have been considered to be the most likely agent behind the cardiotoxic effects, while inflammatory mediators are able to trigger cardiac cell death (Henriksen, 2018). With respect to cardiotoxicity, there are three types: acute, which occurs right after chemotherapy, early, occurring <1 year of therapy, and late, which takes place over several years. These three types were first discovered in 1980, and are summarized in Figure 1 (Zamorano et al., 2016): 1) Acute cardiotoxicity is often reversible, possibly even after a single dose or course of treatment, and symptoms generally appear 14 days after the conclusion of treatment, 2) Early-onset chronic cardiotoxicity manifests as dilated-hypokinetic cardiomyopathy, and progresses towards HF in <1 year, and 3) Late-onset chronic cardiotoxicity occurs multiple years post-anthracycline therapy.

Among breast cancer patients, 1% have acute cardiotoxicity, which is often reversible and presents as electrocardiographic (ECG) abnormalities, transitory left ventricular failure, and supraventricular arrhythmias. Additionally, a consensus joint statement defined LVEF reduction by 10%–53% to be cancer treatment-attributable cardiac dysfunction (Plana et al., 2014). Moreover, a prospective study of 2,625 individuals found that total cardiotoxicity incidence was 9%, with 98% of cardiotoxicity cases being asymptomatic and occurring <1-year post-chemotherapy, plus 82% of patients recovered overall, with 71% having partial, and 11% having complete ECG recovery (Cardinale et al., 2015). Therefore, early detection and treatment of anthracycline-associated cardiac dysfunction with HF medications would be greatly beneficial, as patients frequently have good functional recovery. On the other hand, HF would be difficult to treat if the identification of dysfunction is delayed after its onset (Cardinale et al., 2010). In fact, some other trials have noted that 30%–35% of patients on anthracycline-based regimens had significantly higher blood troponin levels; this increase occurs prior to the onset of more common indicators of cardiac toxicity, such as LV failure (Cardinale et al., 2004; Lipshultz et al., 1997), suggesting that it could serve as a potential early indicator for CRCT. Although troponin has been emphasized as an early indicator, galectin-3, circulating microrna, is an emerging biomarker for CRCT. An important factor in the development of cardiac fibrosis, inflammation, and poor ventricular remodeling—all indicators of the advancement of heart failure—is Gal-3, a lectin released by activated macrophages. According to research on CRCT, anthracycline therapy may result in a rise in serum Gal-3 levels, which is linked to the severity of left ventricular dysfunction that follows (Boer et al., 2010). Research on the possible modulation of Gal-3 by exercise is ongoing. Structured chronic exercise training has been demonstrated to reduce the pro-fibrotic signaling pathway represented by Gal-3 in various cardiac populations, indicating that exercise can reverse the process of maladaptive remodeling, even though acute endurance exercise may momentarily elevate Gal-3 (Kröpfl et al., 2023; Hättasch et al., 2014). Small and stable non-coding Rnas, circulating mirnas offer a lot of promise as early warning biomarkers for CRCT that are both sensitive and specific. Anthracycline exposure often results in an increase in the genes miR-1, miR-133a, and miR-208a, which are implicated in muscle cell integrity and stress response (Ruggeri et al., 2018). It has been demonstrated that exercise training dynamically changes the circulating miRNA profile. Specific C-mirnas respond to both acute and long-term exercise in dose-dependent ways. This “miRNA characteristic” brought on by exercise is thought to be a component of the molecular process underlying cardiovascular adaptation and defense (Baggish et al., 2011). Consequently, tracking these miRNA alterations may offer a fresh method for impartially assessing the biological effectiveness of exercise therapy in reducing CRCT.

### HER2 blockers (ex. trastuzumab, pertuzumab)

HER2 is a critical predictive and prognostic indicator in breast cancer, as its overexpression is linked to poorer prognoses (Slamon et al., 1989); ∼15–30% of all breast cancers are HER2^+^ (Gonzalez-Angulo et al., 2009; Zhang and Yu, 2013; Vu and Claret, 2012). It is able to be selectively targeted by the recombinant humanized monoclonal antibody trastuzumab, which was first approved in 1998 as a first-line therapy for HER2^+^ recurrent metastatic breast cancer. However, ∼2–7% of trastuzumab-treated individuals have reported trastuzumab-induced cardiotoxicity (TIC) (Seidman et al., 2002). Therefore, the increase in cardiotoxicity risk limits the applicability of findings demonstrating that a combination of trastuzumab and anthracyclines increases HER2^+^ breast cancer survival rates by 35% (Slamon et al., 2011; Perez et al., 2008a). Furthermore, breast cancer recurrence could occur if trastuzumab administration is halted when treating TIC.

A number of studies have proposed multiple cellular and molecular pathways for TIC etiology and pathogenesis, such as autophagy suppression, cellular metabolic changes, as well as neureglin-1 (NRG-1)/HER signaling pathway inhibition (Wu et al., 2022). Its severity also seems to be significantly influenced by prior exposure to anthracyclines (Naumann et al., 2013; Leung and Chan, 2015), which may be due to trastuzumab inhibiting the HER2 pathway, subsequently exacerbating anthracycline-caused oxidative stress (Anjos et al., 2021). However, TIC has been found in clinical studies to be reversible and dose-independent, and not every patient receiving treatment develops this side-effect (Chen et al., 2012). Nevertheless, some TIC symptoms include atrial flutter, sick sinus node syndrome, HF, LV dysfunction, and arrhythmia onset (Karaca et al., 2018), of which the most serious and prevalent ones are HF and LV dysfunction (Zamorano et al., 2016). Indeed, the NCCTG N9831 (Alliance) long-term cardiac safety analysis revealed that individuals receiving trastuzumab had higher 6-year cumulative incidence of congestive HF or cardiac mortality, though most individuals had LVEF recovery (Advani et al., 2016). Another observational study found that the greatest cardiomyopathy incidence was observed among invasive breast cancer patients who received a combination of trastuzumab and anthracycline over an 8-year period, comprising 3.5% of 12,500 women (Bowles et al., 2012), and that trastuzumab-only patients had higher cardiomyopathy incidence than anthracycline-only therapy. These findings were in line with those from Thavendiranathan et al. (2016), who noted that estimated cumulative incidences for major cardiac events was higher in trastuzumab-only than anthracycline-only treatment groups. All of these findings thus suggest that trastuzumab and TIC may be a greater concern for treating breast cancer patients than anthracycline.

Other recombinant humanized monoclonal antibodies include pertuzumab, which also targets HER2, where it interferes with oncogenic signaling and stops HER2 dimerization with HER3 (Chung and Lam, 2013). A pooled study of 14 clinical trials found that the risk of asymptomatic LV systolic dysfunction was 6.9% for pertuzumab alone, and 6.5% when combined with trastuzumab (Lenihan et al., 2019); HF incidence, though, was 0.3% for pertuzumab alone, and 1.1% for pertuzumab + trastuzumab. On the other hand, a 2021 comprehensive review highlighted that pertuzumab raises the risk of symptomatic HF, but not asymptomatic LV dysfunction, or LV dysfunction with minor symptoms (Alhussein et al., 2021). Therefore, further analyses should be conducted regarding the precise effects of pertuzumab on the cardiac health of breast cancer patients. Similar analyses should also be conducted on the trastuzumab compound T-DM1 (ado-trastuzumab emtansine), which combines transtuzumab with a cytotoxic microtubule inhibitor, enabling targeted delivery of that inhibitor to HER2-overexpressed cells (Verma et al., 2012; Minckwitz et al., 2019). Using tumor-specific targets, this antibody-drug-conjugate (ADC) avoids non-target effects while selectively delivering extremely harmful medicines to cancer cells (Marmé, 2022). Adcs are transported into cells by lysosome-connected early and late endosomes. Certain linker approaches determine when payloads may be released. Certain ADCs work by releasing cytotoxic medications into the tumor microenvironment outside of the cells. They can discharge these poisons within the cells through cell-permeable payloads or exit the cells by releasing payloads outside the cells prior to internalization. When the target antigen is either not expressed at all or just partially expressed, the bystander killing effect can kill nearby cells. This effect may be particularly useful for cancers with heterogeneous antigen expression (Marmé, 2022; Filho et al., 2021). T-DM1 has been approved for use for metastatic and adjuvant treatments, but its cardiotoxicity, as well as that of other trastuzumab conjugates in clinical settings, has not been fully defined, though one pooled study observed cardiac event occurrence among T-DM1-treated individuals being 3.3%, and the most common cardiotoxicity manifestation was asymptomatic LVEF decrease (Pondé et al., 2020).

### Other breast cancer treatment agents

Other breast cancer chemotherapy medications include cyclophosphamide, which has been used for adjuvant chemotherapy regimens; it has been associated with elevated risk for abrupt HF failure at a cumulative dosage of 150 mg/kg, with likelihood of 7%–33% (Dhesi et al., 2013). Additionally, fluorouracil (5-FU) and capecitabine may increase arrhythmia and myocardial infarction risk (Sara et al., 2018), while the combination of taxanes and anthracyclines could severely harm cardiac muscle (Ojima et al., 2016; Jordan and Wilson, 2004). Indeed, a report indicated that 2.3%–8% of taxane-treated individuals had LV dysfunction (Schlitt et al., 2014). For vinorelbine, though, only ∼1.2% of patients had cardiac incidents (Lapeyre-Mestre et al., 2004). A mixed picture is present for tamoxifen and aromatase inhibitors (AI), in which a meta-analysis of 60,000 breast cancer patients found that tamoxifen was associated with lowered cardiac risk, but no such changes were present for AI (Khosrow-Khavar et al., 2017). On the other hand, another retrospective review of 18,000 breast cancer patients found that AI was linked to a higher HF, mortality risk (Khosrow-Khavar et al., 2020), as well as cardiac events, such as myocardial infarction and ischemic stroke, while tamoxifen was linked to increased thromboembolic events (Fisher et al., 2005).

For treating metastatic breast cancer, another approved medication is lapatinib, a reversible inhibitor of EGFR and HER2 tyrosine kinases; its associated cardiotoxicity is also reversible and less common than TIC, though ∼1.5% of individuals have LV systolic dysfunction as a symptom (Perez et al., 2008b). This cannot be said, though, for cyclin-dependent kinase inhibitors, such as palbociclib, ribociclib, and abemaciclib, which have been administered in conjunction with other treatments to treat advanced and metastatic breast cancer. These drugs have been linked to numerous cardiotoxicity manifestations, such as venous thrombo-embolism and QTc prolongation. More specifically, ribociclib has been linked to QT interval prolongation, such as in the MONALEESA-2 study, where 3.3% of patients, receiving a 600 mg dose, had average QTc intervals >480 m (Hortobagyi et al., 2016); however, these intervals shortened when lowering or eliminating drug administration. Furthermore, a number of clinical investigations found that ribociclib is associated with 5%, and palbociclib with 1.5% risk, of thrombo-embolic events (Hortobagyi et al., 2016; Finn et al., 2016; Finn et al., 2015), while combining those two drugs with endocrine therapy increased venous thromboembolism 3.5-fold, according to a meta-analysis of phase II and III trials (Thein et al., 2018).

For advanced triple-negative breast cancer, immune checkpoint inhibitors, such as pembrolizumab and atezolizumab (Kwapisz, 2021), have been applied as they are able to block PD-L1 (programmed death ligand 1). These inhibitors have been linked to various cardiovascular events, such as arrhythmias, coronary artery disease, vasculitis, and pericarditis, though the most prevalent, accounting for 45% of such events, is myocarditis. It is worth noting that this myocarditis, ranging from moderate to full-blown, is largely reversible (Ball et al., 2019) (Table 1).

**TABLE 1:** Cardiotoxicity effects mediated by various chemotherapy drugs.

Chemotherapeutics	Cardiotoxicity pathogenesis	References
Anthracyclines	• Altering DNA structure and inhibiting its interactions with other proteins→prevent cell division • Generate inflammatory mediators that trigger cardiac cell death	Group et al. (2012), Abdel-Qadir et al. (2017), Cardinale et al. (2015), Srikanthan et al. (2017), Khouri et al. (2012), Hoff et al. (1979), Author AnonymousEarly Breast Cancer Trialists’ Collaborative Group EBCTCG (2023), Friedman et al. (1978), Herrmann et al. (2014), Armenian et al. (2017), Manrique et al. (2017), Broder et al. (2008), Henriksen (2018)
HER2 blockers	• Trastuzumab: autophagy suppression, cellular metabolic changes, NRG-1/HER signaling pathway inhibition • Pertuzumab: Interferes with oncogenic signaling, stops HER2-HER3 dimerization • T-DM1 (ado-trastuzumab emtansine): Combination of trastuzumab with cytotoxic microtubule inhibitor DM1 enables targeted delivery to HER2 over-expressed cells	Slamon et al. (1989), Gonzalez-Angulo et al. (2009), Zhang and Yu (2013), Vu and Claret (2012), Seidman et al. (2002), Slamon et al. (2011), Perez et al. (2008a), Wu et al. (2022), Naumann et al. (2013), Leung and Chan (2015), Chen et al. (2012), Karaca et al. (2018), Advani et al. (2016), Thavendiranathan et al. (2016), Chung and Lam (2013)
Other agents	• Cyclophosphamide: Elevated risk of abrupt cardiac failure associated with cumulative dosage of 150 mg/kg • 5-FU and capecitabine: Increased arrythmia and myocardial infarction risk • Taxanes and anthracyclines: Severe heart muscle damage	Dhesi et al. (2013), Sara et al. (2018), Ojima et al. (2016), Jordan and Wilson (2004), Schlitt et al. (2014), Lapeyre-Mestre et al. (2004), Khosrow-Khavar et al. (2017), Khosrow-Khavar et al. (2020), Fisher et al. (2005), Perez et al. (2008b), Hortobagyi et al. (2016)

### The ability of exercise to improve cardiotoxicity

Exercise has been acknowledged as a safe, efficient supportive therapy for breast cancer survivors (Schmitz et al., 2010), as well as being a feasible non-pharmacological strategy for controlling multiple cardiovascular risk factors (Lavie et al., 2015; Al-Mallah et al., 2018). Indeed, a number of meta-analyses have emphasized the key role of exercise in cancer monitoring and illness recurrence (Ibrahim and Al-Homaidh, 2011), as well as its positive impacts on physiological and psychological outcomes, both during or after treatment (Furmaniak et al., 2016; Lahart et al., 2018). Additionally, evidence suggests that physical exercises (PE), practiced before, during, or after breast cancer treatments, could increase cardiac tolerance against numerous cardiotoxic agents, thereby improving several functional, subclinical, and clinical parameters. The cardioprotective effects of exercise, at the molecular level, have mainly been associated with exercise-induced increases in stress response proteins heat-shock proteins (HSP) 60 and 70, as well as antioxidant activity, such as for superoxide dismutase (SOD) and glutathione (GSH), coupled with lowered lipid peroxidation and pro-apoptotic protein expression, such as Bax (decreased Bax to Bcl-2 ratio). Cardio-protection could also be potentially attributed to the preservation of myosin heavy chain (MHC) isoform distribution (Tranchita et al., 2022). The impact of exercise on cardiovascular diseases in breast cancer patients is a current hot topic. Considering that cardiovascular diseases are the main cause of death for patients who survive for 50–90 years after treatment (Patnaik et al., 2011), this is still worth our further exploration.

Moreover, exercise, plus other lifestyle modifications have been shown to significantly lower breast cancer mortality and recurrence risks (Cannioto et al., 2023), in which breast cancer patients who engaged in moderate physical exercise lowered their mortality likelihood by 60%, compared to those who did not (Cannioto et al., 2021; Chen et al., 2022). In light of such observations, the America College of Sports Medicine recommended that cancer patients should engage in 150 min moderate-intensity aerobic, 75 min intense aerobic, or a comparable mix of exercises weekly (Campbell et al., 2019), while the Consensus Statement from the International Multidisciplinary Roundtable on Exercise Guidelines for Cancer Survivors, last revised in 2019, suggested that cancer survivors engage in moderate-intensity exercise for ≥90 min/week (Campbell et al., 2019). Aside from increasing breast cancer survival likelihood, Howden et al. also found that patients who enganged in more physical activities pre-chemotherapy had lowered cardiotoxicity (Howden et al., 2019). Therefore, devising targeted exercise regimens requires an initial assessment of clinical parameters, which allows for the stratification of patients sharing common conditions, despite their heterogeneity, into homogeneous subgroups (ex. “pheno-groups”) (Scott et al., 2018). As a result, phenotyping is required to identify subtype-dependent treatment strategies (Kyodo et al., 2023), meaning that evaluating “pheno-groups” within breast cancer patient populations, based on biological (ex. Age, body mass index, muscle mass), and cancer-associated clinical characteristics (ex. Diabetes, hypertension, obesity), as well as medications (ex. Insulin, beta-blockers), physical activity history, and cardio-respiratory fitness levels, could provide a more precise view of the impact of physical exercise during chemotherapy on cardiac function, despite population heterogeneity (Linhares et al., 2024). In relation to breast cancer patients’ diets, a randomized clinical study showed that breast cancer survivors who ate a Mediterranean diet, which is abundant in fruits and vegetables, had higher blood antioxidant capacity (vitamin C and coenzyme Q10) (Skouroliakou et al., 2018). The results of these research provide credence to the idea that eating a balanced diet high in whole and plant foods might reduce oxidative stress and boost the body’s antioxidant reserves. It is crucial to keep in mind that dietary antioxidants inhibit too many ROS and, therefore, tumor processes including angiogenesis and metastasis (Ilghami et al., 2020). Nevertheless, encouraging exercise is important for all breast cancer patients (Patel and Rees-Punia, 2022), even accounting for patient heterogeneity in terms of clinical, morphological, physiological and medication conditions, which could result in different hemodynamic responses after applying a given exercise protocol. Owing to this heterogeneity, a comprehensive review, including meta-analyses, recommended that control over potential sources of variability in exercise programs, as well as in assessing cardiotoxicity, should be improved, albeit this is coupled by the observation of encouraging results for exercise-mediated cardio-protection (Ghignatti et al., 2021).

Exercise advantages in CRCT models have been examined in several pre-clinical studies, particular with respect to aerobic exercise (AE) and resistance training (RT). For instance, one study found that both AE and RT exhibited cardioprotective effects, via their abilities to reduce DOX-induced oxidative stress and apoptosis (Varghese et al., 2021). This was in line with Wonders et al., in which a single treadmill jogging session for rats, 24 h pre-DOX treatment, reduced cardiac lipid peroxidation, a sign of oxidative stress (Wonders et al., 2008). This lowered oxidative stress could likely be due to, according to Wang et al., lowered drug penetration into cardiac tissue (Wang et al., 2018), meaning that AE reduces cardiotoxicity during DOX exposure, possibly by altering DOX delivery to myocardial tissue. Additionally, Sequeira et al. observed that AE, combined with DOX treatment, resulted in significantly altered myocardial structures, including decreased fibrosis, along with maintained myofibril integrity and sarcomere organization (Sequeira et al., 2021). Decreased fibrosis could stem from downregulation of fibrosis factor, as identified by Yang et al. (2020), who showed that treadmill exercise prevented DOX-induced cardiac dysfunction by downregulating transforming growth factor (TGF)-β1, phosphorylated extracellular signal-regulated kinase (p-ERK), specificity protein 1 (Sp1), and connective tissue growth factor (CTGF), as well as DOX-stimulated production of IκBα, NF-κB, cyclooxygenase (COX)-2, and interleukin (IL)-8. Exercise is also thought to lessen oxidative stress and apoptosis in breast cancer patients, preserving the function and defense of cardiomyocytes without interfering with cancer therapy (Pfannenstiel and Hayward, 2018; Parry and Hayward, 2018; Schoot et al., 2022; Bigaran et al., 2022; Hall et al., 2019). Exercise may be a useful therapeutic option for cancer in this population, according to research, as it increases VO2 and vascular endothelial function (Beaudry et al., 2018). In order to evaluate the acute effects of exercise on cardiovascular function in patients with breast cancer, Kirkham et al. (2017) divided the participants into two groups: one was instructed to refrain from intense exercise for 72 h prior to chemotherapy, while the other group engaged in a 30-min session of intense aerobic exercise (70% of reserve heart rate) 24 h prior to the first chemotherapy infusion. While both groups showed elevated cardiotoxicity markers, such as cardiac troponin T and amino-terminal of type B natriuretic peptide (NT-proBNP), the exercise group decreased NT-proBNP in comparison to the control group. Only the exercise group reduced diastolic and mean blood pressure, as well as systemic vascular resistance, according to the cardiac outcomes analyses. Within 24–48 h of beginning chemotherapy, the exercise group’s pulse pressure and left ventricular ejection fraction were higher than those of the control group. This data suggests that PE may play a crucial part in controlling risk variables associated with CVD, particularly in women with breast cancer receiving anthracycline therapy (Lee et al., 2019). The following table compares the efficacy of different types of exercise in reducing CRCT induced by different chemotherapy drugs (Table 2).

**TABLE 2:** Comparative analysis: exercise strategies for specific drug-induced toxicity.

Feature	Anthracycline-induced toxicity	Trastuzumab-induced toxicity
Primary Mechanism	Oxidative stress and mitochondrial damage, leading to cardiomyocyte apoptosis and necrosis (Wang et al., 2023; Li et al., 2025)	Blockade of ErbB2 (HER2) survival signaling in cardiomyocytes, causing reversible contractile dysfunction (Foulkes et al., 2023)
Recommended Priority	Aerobic Exercise (foundational) HIIT or Combined Training (theoretical potential) (Foulkes et al., 2023; Zhang et al., 2025)	HIIT or Combined Training (theoretical potential) (Wilson et al., 2023)
Rationale	Foundational for upregulating antioxidant defenses and promoting mitochondrial biogenesis. The steady-state nature provides a safe hemodynamic profile during active treatment (Paraschiv et al., 2025; Po and pov, 2020)	The powerful physiological stimulus may activate alternative cardio-protective signaling pathways (e.g., IGF-1), potentially compensating for the blocked ErbB2 pathway (Wilson et al., 2023)
Evidence and Considerations	Strong evidence from RCTs shows aerobic exercise improves VO2peak, cardiac output, and reduces troponin elevation (Foulkes et al., 2023). HIIT should be used with caution during active treatment	Direct comparative evidence is limited. A dedicated trial at Dana-Farber Cancer Institute is investigating HIIT for patients on chemo, including those likely receiving trastuzumab (Wilson et al., 2023)

Exercise training may also affect cardiomyocyte metabolism through adenosine monophosphate-activated protein kinase (AMPK). PAK1 is activated by various cell surface or intracellular signals, activates the MAPK signal pathway, changes the shape of the cytoskeleton and acts as an oncogene in breast cancer (Torun et al., 2024). Kitani et al. found that in human induced pluripotent stem cell-derived cardiomyocytes (iPSC-CMs) treated with trastuzumab, the pharmacological activation of AMPK promoted glucose absorption, improved mitochondrial respiratory capacity and systolic dysfunction (Kitani et al., 2019), This is also in line with the view of Coven et al., who observed that the increase in AMPK activity was a prominent cardiac adaptation related to exercise in rats (Coven et al., 2003). Based on those findings, exercise could potentially alleviate TIC-linked metabolic impairments and subsequent contractile dysfunction, by increasing AMPK activation. Aside from increased AMPK activity, AE has been found in rodents to upregulate Pparg coactivator (PGC)-1α, a critical mitochondrial biogenesis regulator, whose activity is hampered in trastuzumab-treated iPSC-CMs (Kitani et al., 2019); indeed, rats who underwent vigorous exercise had a 37% increase in mitochondrial density, and 44% increase in cardiac PGC-1α (Tam et al., 2015). Similar benefits, along with that of enhanced mitochondrial capacity, fatty acid oxidation, and glycogen production (Riehle et al., 2014) were also observed in other animal exercise models (Vettor et al., 2014). Based on these findings, even small amounts of exercise could alleviate pathogenic cardiac remodeling, via counteracting the proinflammatory effects of trastuzumab; this was demonstrated in a rat myocardial infarction-induced HF model, where AE reduced plasma tumor necrosis factor-α and IL-6 (Nunes et al., 2013). Anthracyclines mainly induce cardiotoxicity by damaging mitochondria and causing oxidative stress, which results in the death of cardiomyocytes (Narezkina et al., 2021). This major damage mechanism is directly countered by exercise-induced AMPK activation, which aids in restoring redox equilibrium (the cellular balance between oxidants and antioxidants). Moreover, PGC-1α activation is a master regulator of mitochondrial biogenesis, which is the process by which new, healthy mitochondria are created (Taha et al., 2025). The main cause of trastuzumab’s cardiotoxicity is not oxidative stress. Instead, it includes cardiomyocytes’ HER2 signaling being blocked, which is essential for their survival and ability to contract. Unlike anthracycline toxicity, which is dose-dependent, this kind of damage is frequently reversible (Taha et al., 2025).

Regular exercise primarily increases the body’s capacity for adaptation in breast cancer patients by increasing the number of mitochondria. This allows the body to react to oxidative stress more rapidly, reducing cell damage and boosting antioxidant capacity (Genest et al., 1979; Traustadóttir et al., 2012). Physical exercise was linked to decreased levels of DNA oxidation indicators, including F2-isoprostanes and 8hydroxydeoxyguanosine (8-OhdG), and lipid peroxidation, according to various studies (Traustadóttir et al., 2012; Campbell et al., 2010; Schmitz et al., 2008). In addition to helping to prevent sarcopenic obesity and enhance prognosis (Artene et al., 2017), physical activity during AT also improves patients’ tiredness and functional ability (Juvet et al., 2017; Browall et al., 2018). A solid and flexible basis for creating workable rehabilitation plans for breast cancer survivors is provided by current general cardiac rehabilitation (CR) protocols, especially for heart failure (Ali and Mullen, 2025). One of the main components of CR, exercise, is immediately relevant but has to be modified. For patients with breast cancer, this entails accounting for side effects of treatment, such as peripheral neuropathy brought on by chemotherapy, which impairs balance, and being aware of the possibility of lymphedema. Numerous forms of exercise have been shown to improve cardiovascular outcomes for cancer patients (Miyata et al., 2025).

DOX has been noted to cause apoptosis by two ways: redox uncoupling and intrinsic mitochondrial mechanisms (Minotti et al., 2004), which, however, could potentially be prevented by AE, as Ascensao et al. found that AE was able to preserve cardiac mitochondrial chain complexes I and V, preventing DOX-induced mitochondrial activity reductions (Ascensão et al., 2011). Furthermore, moderate endurance training intervention among rats also notably alleviated DOX-induced calcium sensitivity, uncoupled respiration, aconitase activity, and mitochondrial state three respiration alterations (Ascensão et al., 2005). Exercise training also prevented DOX-associated increases in apoptotic protein activity and carbonyl groups in mitochondrial proteins, as well as, according to Kavazis et al. (2010), cardiac mitochondrial ROS production. In terms of cardiac function, rats who voluntarily ran on wheels for 8 weeks pre-treatment had less DOX-associated LV functional losses (Chicco et al., 2005). This is also supported by an echocardiographic study comparing sedentary rats with those who ran in a wheel or treadmill for 10 weeks, and the exercised ones had intact heart function at 10 days post-DOX injection, with fractional shortening only decreasing by 2% and 3%, respectively, after wheel or treadmill exercises, compared to 15% for sedentary rats (Hydoc et al., 2008).

Exercise rehabilitation could also reduce the likelihood of breast cancer patients for developing delayed cardiotoxicity (Miura et al., 1979; Greenland et al., 1999; Cheng et al., 2021), by lowering resting heart rates (RHR). Indeed, Fairey et al. found that post-menopausal breast cancer survivors, after undergoing a 15-week exercise regimen, had RHR reductions by 5.5 beats/min, along with substantial increases in HR reserves (Fairey et al., 2005); these survivors also had substantial HR raises during peak exertion (Courneya et al., 2003). This was supported by Hambrecht et al., who found that RHR dropped by nine beats/min among HF patients after cardiac rehabilitation (Hambrecht et al., 2000a). Overall, HR parameters are reliable measures of cardiac function, and have long been associated with mortality.

As for the feasibility of exercise for cardiac rehabilitation in breast cancer, such individuals are strongly driven to alter their lifestyles, especially in the post-diagnosis, pre-treatment period. In fact, 38.8% of breast cancer survivors would prefer to receive exercise advise pre-treatment, compared to 18.7% during, 21.5% immediately after, and 21.2% ≥ 3 months post-treatment (Jones and Courneya, 2002). Furthermore, studies have found that little as 4 weeks exercise training can considerably enhance cardiovascular function in breast cancer patients with cardiovascular conditions, including hypertension or coronary artery disease (Collier et al., 2008; Hambrecht et al., 2000b). Sixteen weeks of high-intensity interval training (HIIT) was also equally effective in avoiding body mass increases, maintaining cardiorespiratory fitness, increasing muscle strength, and lowering pain sensitivity, among breast cancer patients undergoing chemotherapy (Mijwel et al., 2018). Another HIIT trial found that it had positive effects on cancer-related tiredness, symptoms, and muscular strength, from 12 months to 2 years, after starting chemotherapy (Mijwel et al., 2019) (Bolam et al., 2019). Despite several cardiac rehabilitation programs currently being available for aiding individuals with cardiac issues, though, they do not specifically address the requirements of breast cancer patients, especially as they are at higher risk for cardiotoxicity. Therefore, personalized breast cancer patient exercise regimens should be devised, taking into account the specific medical profile, such as cancer stage, treatment status, and treatment regimen, as well as the psychological state, of each patient (Varghese et al., 2021).

## Conclusion

This paper offers a thorough review of the manifestations and underlying mechanisms behind CRCT in breast cancer patients, as well as summarizing the most recent findings on the benefits of exercise for improving cardiovascular health in this setting. A variety of chemotherapeutic drugs, falling into three broad categories, have been found to be linked to CRCT: anthracyclines, particularly DOX, HER2 inhibitors trastuzumab, pertuzumab, and T-DM1, as well as other medications, such as the alkylating agent cyclophosphamide, cyclin-dependent kinase inhibitors, immune checkpoint, 5-FU, etc. These medications mainly operate by increasing pro-inflammatory cytokine and reactive oxygen species production, leading to cardiomyocyte damage, and subsequently increasing HF risk. CRCT, though, could be alleviated by a number of exercise regimens, particularly AE, RT, and HIIT, all of which are safe and are able to enhance systolic and cardiorespiratory performance among breast cancer patients. However, specifically applying these regimens for breast cancer patients, post-chemotherapy, has not been fully characterized, in terms of the type, frequency, intensity, and timing. Therefore, tailored exercise regimens, which fit the specific breast cancer medical profile, in terms of cancer stage, treatment status, and treatment regimen, as well as psychological state, should be developed to alleviate the adverse cardiovascular effects of chemotherapy.
